# The Association Between Fasting Blood Sugar and Index of Nutritional Quality in Adult Women

**DOI:** 10.3389/fnut.2022.883672

**Published:** 2022-06-24

**Authors:** Farkhondeh Alami, Golsa Khalatbari Mohseni, Mina Ahmadzadeh, Farhad Vahid, Maryam Gholamalizadeh, Mohammad Masoumvand, Soheila Shekari, Atiyeh Alizadeh, Hanieh Shafaei, Saeid Doaei

**Affiliations:** ^1^Department of Nutrition, Faculty of Medicine, Urmia University of Medical Sciences, Urmia, Iran; ^2^Department of Nutrition, School of Allied Medical Sciences, Ahvaz Jundishapur University of Medical Sciences, Ahvaz, Iran; ^3^Department of Clinical Nutrition and Dietetics, Faculty of Nutrition and Food Technology, National Nutrition and Food Technology Research Institute, Shahid Beheshti University of Medical Sciences, Tehran, Iran; ^4^Department of Population Health, Public Health Research, Luxembourg Institute of Health, Strassen, Luxembourg; ^5^Cancer Research Center, Shahid Beheshti University of Medical Sciences, Tehran, Iran; ^6^Department of Nutrition, Faculty of Medicine, Mashhad University of Medical Sciences, Mashhad, Iran; ^7^Department of Nutrition, Science and Research Branch, Islamic Azad University, Tehran, Iran; ^8^Department of Pharmacognosy, Faculty of Pharmacy, Tehran University of Medical Science, Tehran, Iran; ^9^Urology Research Center, Razi Hospital, School of Medicine, Guilan University of Medical Sciences, Rasht, Iran; ^10^Department of Community Nutrition, Faculty of Nutrition and Food Technology, National Nutrition and Food Technology Research Institute, Shahid Beheshti University of Medical Sciences, Tehran, Iran

**Keywords:** index of nutritional quality, fasting blood sugar, dietary intake, glycemic control, medical history

## Abstract

**Aim:**

It's unclear whether diet quality affects glycemic management. The index of nutritional quality (INQ) can examine diets both quantitatively and qualitatively (INQ). Hence, this study aimed to determine whether INQ and fasting blood sugar (FBS) are related among Iranian women.

**Methods:**

This cross-sectional study was conducted on 360 adult Iranian women. Data were collected on the participants' general characteristics, medical history, anthropometric indices, physical activity, and dietary intake. For nutrient intake assessment, a valid food frequency questionnaire (FFQ) was used, and INQ was then calculated using the daily nutrient intake.

**Results:**

After adjusting for age, FBS was significantly inverse associated with INQ for vitamins A (B = −0.193, *p* < 0.01), magnesium (B = −0.137, *p* < 0.01), phosphor (B = −0.175, *p* < 0.01), zinc (B = −0.113, *p* < 0.01), vitamin K (B = −0.197, *p* < 0.01), manganese (B = −0.111, *p* < 0.01) and selenium (B = −0.123, *p* < 0.01). The association between FBS and INQ for Se and Mn was disappeared after further adjustment for gender, body mass index (BMI), menopausal status, and total energy intake.

**Conclusion:**

There was a significant inverse relationship between FBS and the INQ of vitamin A, manganese, phosphor, zinc, vitamin K, magnesium, and selenium. Prospective cohort studies should be conducted to establish a causal relationship between FBS and INQ.

## Introduction

Hyperglycemia is observed in about one-fifth to one-fourth of the adult population in developing countries (1). Hyperglycemia is defined as a fasting blood glucose level of more than 126 mg/dl or a random blood glucose level of more than 200 mg/dl, both of which are common among hospitalized patients (2, 3). The development of hyperglycemia is linked to an increased risk of mortality (4) and infections in hospitalized patients, according to extensive data from observational studies (5). Some studies reported that correction of hyperglycemia with insulin administration reduces hospital complications and decreases mortality in cardiac diseases (6).

Dietary components may have significant effects on the management of hyperglycemia. Recently, several indicators were introduced to evaluate the quality of the diet. For example, glycemic index (GI) is a value used to measure how much specific foods increase blood sugar levels, and foods with a low GI < 50 cause a slower rise in blood glucose concentration compared to an equal carbohydrate amount from high GI foods (7). Evidence suggests that dietary management with a low GI diet improves glycemic control in diabetic patients (7, 8). Although carbohydrate is likely the most significant component of food to affect postprandial glycemia, previous research found that dietary fat and protein have a key role in the glycemic response following the ingestion of carbohydrate (9). Dietary fat can delay hyperglycemic peak responses by slowing glucose absorption (10–12) and dietary protein facilitates glucose clearance by stimulating insulin release (13–16). A decreased incidence of hyperglycemia was linked to a 'healthy traditional' dietary pattern rich in vegetables, grains, and products, fish, and shrimp. Greater consumption of fruits, juice, and alcohol, on the other hand, was linked to a higher risk of hyperglycemia (17–19). Controversial results in relation to hyperglycemia and dietary components were previously observed. For example, some studies reported that fat intake is positively associated with the prevalence of impaired fasting glucose (20, 21). While other studies did not highlight an association between total fat intake and the risk of type-2 diabetes mellitus (T2DM) (22–25).

The index of nutritional quality (INQ) score was created to assess the diet quality and comprises four components: variety, adequacy, moderation, and overall balance (26) and was used in a few studies (27–29). Previous studies in European adults have reported an inverse association between INQ and cardiovascular risk factors, including lipid biomarkers and obesity (30). The incidence of fasting blood sugar disorder was reported to decrease by 75% in men with higher scores of INQ (31). In another study, the patients in the highest tertile of INQ had fewer fasting blood sugar amounts. However, no significant correlations were observed in some studies between dietary quality indices and fasting blood sugar (29). For example, in a cross-sectional study on the correlation between diet quality and glycemic status in patients with type 2 diabetes, no significant correlations were observed between INQ and fasting blood sugar, glycosylated hemoglobin (HbA1c), insulin, and insulin resistance [28]. To the best of our knowledge, no firm association between INQ and FBS has been yet established. So, this study aimed to investigate the association between INQ and FBS in Iranian adults.

## Methods

This cross-sectional study was performed on 360 Iranian adult women in Tehran, Iran. The participants were selected from healthy women referring to the nutrition clinic of Shohadaye Tajrish Hospital, Tehran, Iran. The sample size was calculated using the OPENEPI software and the quantity of odds ratio (OR) acquired in a prior research [19]. The inclusion criteria were willingness to participate in the study, age between 35 and 75 years old, having no history of metabolic syndrome, not suffering from diseases affecting blood sugar, and did not use antihyperglycemic drugs. Participants with alcohol or drug addiction, have weight-related illnesses including specific psychological or neurological disorders, insulin resistance, thyroid disease, liver disease, kidney failure, infectious diseases, history of multiple sclerosis, hypertension, dialysis, and pregnant or lactating women were excluded from the study (*n* = 7). The objectives of the study were explained to the participants, and a written consent form was collected. Data on age, height, weight, and BMI were collected through face-to-face interviews and the amount of physical activity was estimated using a validated International Physical Activity Questionnaire (IPAQ).

### FBS Measurement

Five ml of blood samples were collected from all participants after 10–12 hr of overnight fasting. In order to prevent glycolysis, plasma was isolated up to 1 h after sampling, and blood glucose levels were measured using glucose oxidase and photometry using the colorimetric method GOD-PAP solution (Pars Azmoun, Iran) and RA-1000 auto analyzer.

### Dietary Assessment

A semi-quantitative food frequency questionnaire (FFQ) that has previously been validated in Iran was used to collect the necessary nutritional data [6]. The FFQ consisted of 147 food items with standard serving sizes commonly used by Iranians. Participants were asked to report the frequency of consumption of each food item according to its standard portion size during the last year. Final portion sizes were changed into g/day using household measures based on USDA database with minor modification for the special national foods like breads and the average. Then, the data obtained from these questionnaires were analyzed using nutritionist-IV software (version 4.1; First Databank Division; Hearst) and daily intake of energy and nutrients was calculated.

The INQ score assess variety, adequacy, moderation, and overall balance of the diet, thus it may capture different aspects of diet quality related to under- and over-nutrition (26).

The INQ analyzes foods, meals, and diets quantitatively and qualitatively and compares people's dietary intakes extracted from the FFQ with the recommended standards. It modifies the effect of total energy intake and provides accurate estimations of individual intake (32). The following nutrients were used in computing INQ: vitamin A, riboflavin, vitamin C, vitamin D, vitamin K, vitamin B6, thiamin, niacin, biotin, folate, vitamin B12, vitamin E, zinc (Zn), iron, copper, manganese (Mn), phosphor (P), manganese (Mg), and selenium (Se).

The INQ for each nutrient was then calculated as the ratio of the amount of nutrient consumed per 1,000 kcal per day to the recommended dietary allowance of the RDA for nutrients per 1,000 kcal (33). In cases where the RDA for specific nutrients was not defined, adequate intake (AI) values were used. Individuals whose average daily energy intake was reported to be <800 or >4,200 kcal or who did not consume more than 70 food items (>40% of items in the food frequency questionnaire) were excluded from the study.

### Statistical Analysis

To compare different variables in individuals with normal and high FBS profiles, independent *t*-test and chi-square methods for quantitative and qualitative variables were used, respectively. A multiple linear regression method was used to investigate the association between fasting blood sugar and the INQ after adjusting for age (model 1), age, gender, BMI, menopausal status, and total energy intake (model 2). The SPSS software version 23 was used for statistical analysis and the probability level of *P* < 0.05 was considered statistically significant.

## Results

The general characteristics of the participants are presented in Table 1. People with higher FBS (*n* = 134) had significantly higher age (52.9 ± 8.7 vs. 47.40 ± 7.4, *P* = 0.001), weight (74.74 ± 11.8 vs. 69.18 ± 10.8, *P* = 0.001) and BMI (30.65 ± 4.17 vs. 28.30 ± 4.08, *P* = 0.001) compared with the participants with normal FBS (*n* = 219). No significant difference was observed between the two groups regarding height, physical activity, smoking, and using alcohol.

**Table 1:** Characteristics of the participants with high and normal levels of fasting blood sugar.

**Parameters**	**Normal FBS** **(*n* = 219)**	**High FBS** **(*n* = 134)**	**P**
Age (y)	47.40 (± 7.4)	52.9 (± 8.7)	0.001
Height (cm)	156.292(± 5.6)	155.970(± 5.3)	0.590
Weight (kg)	69.187(± 10.8)	74.746(± 11.8)	0.000
Body mass index (BMI) (kg/m^2^)	28.3012(± 4.08)	30.6590(± 4.17)	0.000
Physical activity (kcal/kg/h)	1.5646(± 1.5)	1.3769(± 1.4)	0.257
Smoking *n* (%)	1(0.5%)	2(1.5%)	0.56
Using alcohol *n* (%)	2 (0.9%)	1(0.7%)	0.68

Table 2 shows the association between dietary intake among people with normal and high levels of FBS. People with higher FBS (*n* = 134) had significantly higher intake of thiamin (2.51 ± 1.06 vs. 2.05 ± 0.63, P=0.015), riboflavin (2.48 ± 1.55 vs. 1.95 ± 0.82, *P* = 0.046), niacin (25.37 ± 8.60 vs. 22.18 ± 5.95, *P* = 0.051), vitamin B6 (1.93 ± 0.87 vs. 1.60 ± 0.51, *P* = 0.032), folate (704.06 ± 205.20 vs. 612.48 ± 194.57, *P*= 0.051), vitamin B12 (4.41 ± 4.43 vs. 2.96 ± 1.41, *P* = 0.028), biotin (33.45 ± 16.15 vs. 26.67 ± 10.55, *P* = 0.024), P (1,567.48 ± 1,094.6 vs. 1,217.70 ± 492.78, *P* = 0.047), selenium (105.31 ± 45.22 vs. 85.69 ± 26.28, *P* = 0.014) compared with the participants with normal FBS.

**Table 2:** Dietary intakes among people with normal and high levels of fasting blood sugar.

**Parameters**	**Normal FBS**	**High FBS**	**P**
Total energy intake (Kcal/d)	2,473.36 (± 703.65)	2,869.39 (± 997.91)	0.039
Protein (gr/d)	75.67 (± 27.52)	92.10 (± 46.31)	0.45
Carbohydrate (gr/d)	365.51 (± 96.05)	420.47 (± 133.72)	0.34
Fat (gr/d)	84.86 (± 36.46)	96.91 (± 44.66)	0.191
Cholesterol (mg/dl)	216.19 (± 110.84)	254.93 (± 137.90)	0.170
Saturated fatty acids (mg/dl)	25.64 (± 11.97)	31.124 (± 21.40)	0.137
Mono unsaturated fatty acids (mg/dl)	29.292413 (± 14.26)	31.097204 (± 12.94)	0.576
Poly unsaturated fatty acids (mg/dl)	19.653896 (± 10.29)	19.293867 (± 7.65)	0.871
PFA3 (mg/dl)	1.241493 (± 0.64)	1.259295 (± 0.514)	0.899
PFA6 (mg/dl)	6.074097 (± 8.48)	5.141801 (± 6.17)	0.608
Sodium (IU/L)	5,115.400291 (± 2302.7)	5,934.210146 (± 2656.7)	0.150
Potassium (IU/L)	3,652.394859 (± 1477.40)	4,298.316661 (± 1958.69)	0.096
Vitamin A (IU/L)	505.368241 (± 288.28)	444.731172 (± 209.94)	0.277
Beta-carotene (IU/L)	2,924.71 (± 1354.95)	3,212.54 (± 2216.34)	0.464
Alpha-carot (IU/L)	438.97 (± 376.75)	595.76 (± 691.5)	0.183
Lutein (IU/L)	1,573.75 (± 904.35)	1,541.09 (± 742.75)	0.869
Beta-cryptoxanthin (IU/L)	322.25 (± 201.73)	284.90 (± 163.59)	0.400
Lycopene (IU/L)	7,173.65 (± 4313.99)	7,843.467731(± 4,733.68)	0.519
Vitamin C (IU/L)	138.61 (± 138.61)	156.78 (± 156.78)	0.401
Vitamin E (IU/L)	17.96 (± 14.43)	17.546 (± 9.76)	0.892
Alpha-tocopherol (IU/L)	11.841 (± 9.40)	11.48 (± 6.70)	0.858
Thiamin (IU/L)	2.05 (± 0.637)	2.51(± 1.06)	0.015
Riboflavin (IU/L)	1.957 (± 0.829)	2.48 (± 1.55)	0.046
Niacin (IU/L)	22.188 (± 5.95)	25.37 (± 8.60)	0.051
Vitamin B6 (IU/L)	1.60 (± 0.51)	1.93 (± 0.87)	0.032
Folate (IU/L)	612.48 (± 194.57)	704.06 (± 205.20)	0.051
Folate (IU/L)	755.47 (± 237.27)	856.65 (± 301.00)	0.098
Vitamin B12 (IU/L)	2.96 (± 1.41)	4.41 (± 4.43)	0.028
Biotin (IU/L)	26.67 (± 10.55)	33.45(± 16.15)	0.024
Pantothenic (IU/L)	4.71 (± 1.81)	5.61 (± 3.00)	0.092
Vitamin K (IU/L)	127.47 (± 56.87)	118.18 (± 51.52)	0.455
Phosphor (IU/L)	1,567.48 (± 1,094.6)	1,217.70 (± 492.78)	0.047
Magnesium (IU/L)	401.01 (± 169.82)	344.61 (± 131.99)	0.099
Zinc (IU/L)	11.77 (± 6.19)	10.54 (± 5.73)	0.372
Copper (IU/L)	1.79 (± 0.51)	1.97 (± 0.64)	0.167
Manganese (IU/L)	6.06 (± 2.93)	5.18 (± 1.80)	0.091
Selenium (IU/L)	105.31 (± 45.22)	85.69 (± 26.28)	0.014
Iron (mg/dl)	2,956.16 (± 1,620.08)	3,584.31 (± 1,980.78)	0.126
Chromium (IU/L)	014 (± 0.047)	026 (± 0.104)	0.442
Fiber-t (mg/dl)	27.41 (± 10.74)	30.073 (± 11.58)	0.302
Fiber-s (mg/dl)	1.02 (± 4.29)	1.07 (± 0.92)	0.768
Fiber-is (mg/dl)	11.53 (± 6.62)	5.59 (± 4.29)	0.484
Crude-fiber (mg/dl)	11.128 (± 6.82)	11.53 (± 6.62)	0.797
Sugar-t (mg/dl)	128.49 (± 46.73)	150.25 (± 70.52)	0.096
Glucose (mg/dl)	20.30 (± 8.10)	22.096 (± 9.33)	0.369
Gal (mg/dl)	2.69 (± 2.42)	5.09 (± 10.66)	0.114
Fructose (mg/dl)	25.98 (± 10.26)	27.67 (± 11.62)	0.499
Sucrose (mg/dl)	46.40 (± 21.12)	50.49 (± 29.76)	0.471
Lactose (mg/dl)	10.13 (± 7.12)	17.21 (± 28.84)	0.088
Maltose (mg/dl)	2.73 (± 1.23)	3.11 (± 1.68)	0.235
Caffeine (mg/dl)	161.00 (± 94.46)	200.91 (± 117.54)	0.095

Table 3 presents the association between FBS and the INQ of nutrients. There was a significant inverse association between FBS and the INQ of vitamin A (B = −0.193, *P* < 0.01), Magnesium (B = −0.137, *P* < 0.01), P (B = −0.175, *P* < 0.01), zinc (B = −0.113, *P* < 0.01), vitamin K (B = −0.197, *P* < 0.01), Mn (B = −0.111, *P* < 0.01) and Se (B = −0.123, *P* < 0.01) after adjustment for age (model 1). The association between the FBS and INQ of Se and Mn were disappeared after further adjustment for gender, BMI, menopausal status, and total energy intake (Model 2).

**Table 3:** The association between the index of nutritional quality (INQ) of the nutrients and FBS.

**INQ**	**Model 1** ^a^		**Model 2** ^a^	
	**B**	***P*** **-value**	**B**	***P*** **-value**
Vitamin A (IU/L)	−0.193	<0.01	−0.227	<0.01
Mg (IU/L)	−0.137	<0.01	−0.153	<0.01
P (IU/L)	−0.175	<0.01	−0.236	<0.01
Zn (IU/L)	−0.113	0.02	−0.192	<0.01
Copper (IU/L)	−0.018	0.72	0.088	0.16
Mn (IU/L)	−0.111	0.02	0.057	0.37
Se (IU/L)	−0.123	<0.01	−0.097	0.12
E (IU/L)	−0.017	0.71	0.020	0.74
B1 (IU/L)	−0.032	0.50	−0.057	0.36
B2 (IU/L)	0.072	0.13	0.064	0.31
B3 (IU/L)	0.003	0.94	0.049	0.43
B6 (IU/L)	0.051	0.28	0.033	0.60
B9 (IU/L)	0.027	0.57	0.047	0.45
B12 (IU/L)	0.020	0.67	0.038	0.54
B5 (IU/L)	0.058	0.22	0.132	0.05
Biotin (IU/L)	−0.016	0.73	−0.010	0.87
Vitamin C (IU/L)	0.051	0.28	0.055	0.38
Vitamin D (IU/L)	0.037	0.43	−0.005	0.93
Vitamin K (IU/L)	−0.197	<0.01	−0.165	<0.01

a *First model adjusted for age and the second model adjusted for age, gender, BMI, menopausal status, and total energy intake*.

## Discussion

The present study investigated the association between FBS and INQ in adult women. There was a significant difference between the INQ of vitamin A, Mg, zinc, vitamin K, Mn, and selenium with FBS after adjustments for age, gender, BMI, menopausal status, and total energy intake.

Similar to this study, some studies found that vitamin A has both antioxidant and antihyperglycemic potential and, therefore, can be considered a hypoglycemic factor (34, 35). Vitamin A can impact T2DM pathogenesis through several potential molecular mechanisms, including chelation of oxide radicals, improves insulin sensitivity, and beta-cell regeneration (36). Shidfar et al., on the other hand, discovered no correlation between FBS and vitamin A in 48 diabetes mellitus type 1 (DMTI) patients (37). Jafarirad et al. showed in another trial that vitamin A had no significant influence on lipid profiles, FBS, or liver enzymes (38). The most likely explanation for these discrepancies is that the current research employed an assessment of nutritional quality rather than the quantity of nutrients consumed (34).

In line with this study, some studies reported the beneficial effects of Mg on glycemic control in individuals with T2DM (39–42). While, other studies showed no significant effects of Mg on T2DM (39, 40). The improvement in the glycemic control indicators after Mg therapy could be explained by different mechanisms, including the influence of Mg on insulin receptor activity through enhanced tyrosine kinase phosphorylation (43, 44). There was the possibility that Mg could help facilitate the translocation of glucose transporter number 4 (GLUT 4) to the cell membrane caused by the activation of tyrosine–kinase in the presence of Mg (45).

Regarding the association of FBS and dietary phosphorus, Fang et al. reported that the serum level of phosphate in the type 2 diabetic group was significantly lower than that in the control group (46). Besides, Duan et al. demonstrated that higher urinary phosphorus excretion was associated with decreased risk of T2DM (47). However, other research found that phosphorus had no significant influence on T2DM (48). For instance, one research discovered that diabetic rats also had increased plasma phosphorus amounts (49). The reason for the difference in results may be related to the study population and some of which were performed on animals (49). Phosphorus concentration is a determining factor in regulating the metabolism and rate of oxygen consumption. In diabetes, the highest oxygen consumption is associated with the lowest concentration of phosphorus (50).

In terms of the association between of FBS and Zn, a negative association was found between serum Zn with FBS. Low Zn levels were reported to be associated with poor glycemic control and poor glycemic control is a strong predictor of Zn deficiency (51). Another study reported that there is no definite cause-and-effectrelationship between Zn and the level of FBS (52). The reduced concentration of Zn in T2DM was indicated by Saharia and Goswami (53), which was in line with the present study. Al-Maroof and Al-Sharbatti (54) also reported that Zn levels were lower in diabetic patients compared to the controls, and a strong negative relationship was found between glycosylated hemoglobin levels of diabetic patients with their serum Zn levels. Zn has an important role in the utilization of glucose by muscle and fat cells (55). Zn acts as a cofactor for intracellular enzymes involved in protein, lipid, and glucose metabolism (55). Zinc is required for the stability of insulin hexamers and the hormone's pancreatic storage (56).

Similar with the results of the present study, high circulating levels of vitamin K were reported to be related to a lower risk of the high amount of FBS (57). Some studies found a lower risk for diabetes mellitus in people with higher vitamin K intakes (58, 59). Rees et al. (60) in a systematic review of studies that evaluated the association of vitamin K deficiency with T2DM concluded that there is no evidence of an effect of vitamin K and a higher level of FBS. The differences in the obtained results can be related to the study population since some studies have been done on diabetics and some others on healthy people. Vitamin K has been shown to reduce insulin resistance by inhibiting inflammation. Vitamin K may inhibit the generation of IL-6 (Interleukin 6) in lipopolysaccharide-induced inflammatory models (61, 62). Moreover, high plasma Vitamin K concentrations were associated with decreased concentrations of inflammatory markers TNF-α (Tumor necrosis factor alpha) and IL-6 (63). In a study that assessed the status of fat-soluble vitamins in patients with chronic pancreatitis, the results indicated that the serum concentrations of fat-soluble vitamins were decreased in these patients (64).

Tan et al. revealed that pregnant women with impaired glucose tolerance (IGT) or gestational diabetes mellitus (GDM) had lower blood selenium levels and discovered an inverse association between FBS and serum selenium levels (65), which was in line with the present study. However, the evidence for a link between selenium and GDM is inconsistent, with other research finding no correlation between selenium concentration and FBS level (66, 67).

Moreover, a higher intake of manganese was frequently reported to be associated with a lower level of FBS (68–70). However, another study found that both low and high levels of plasma manganese were associated with higher levels of FBS (71). Manganese plays significant roles in multiple physiological functions, including glucose and lipid metabolism, insulin production, and insulin secretion. Manganese deficiency leads to impaired glucose tolerance and increased risk of metabolic syndrome through impaired glucose and lipid metabolism (72, 73).

To the best of our knowledge, few studies investigated the association between FBS and the quality of diet. The strengths of the present study were its acceptable sample size and using the index of nutritional quality. However, the results may be affected by some limitations. This study was a cross-sectional study, which could not explain the causal relationship. Future prospective studies should be performed to establish a causal relationship between FBS and the INQ in adult women. Moreover, this study was performed on young women and cannot be generalized to the public. In addition, the semi-quantitative FFQ is not the best indicator to know the amount of micronutrients that are being ingested and, probably, it would be more indicated to carry out an intake analysis with double weighing in future studies.

The present study provides the first evidence for an association between FBS and the INQ in adult women. According to the findings of the study, there was a significant inverse association between FBS and the INQ of vitamin A, Mg, P, zinc, vitamin K, Mn, and Se. Prospective cohort studies should be performed to establish a causal relationship between FBS and INQ in adult women.

## Data Availability Statement

The raw data supporting the conclusions of this article will be made available by the authors, without undue reservation.

## Ethics Statement

The studies involving human participants were reviewed and approved by IR.SBMU.nnftri.Rec.1400.049. The patients/participants provided their written informed consent to participate in this study.

## Author Contributions

FA, MG, SD, GM, MA, HS, AA, MM, and SD designed the study, involved in the data collection, analysis, and drafting of the manuscript. SD and FV were involved in the design of the study, analysis of the data, and critically reviewed the manuscript. All authors read and approved the final manuscript.

## Funding

The research was financially supported by Shahid Beheshti University of Medical Sciences, Tehran, Iran.

## Conflict of Interest

The authors declare that the research was conducted in the absence of any commercial or financial relationships that could be construed as a potential conflict of interest.

## Publisher's Note

All claims expressed in this article are solely those of the authors and do not necessarily represent those of their affiliated organizations, or those of the publisher, the editors and the reviewers. Any product that may be evaluated in this article, or claim that may be made by its manufacturer, is not guaranteed or endorsed by the publisher.

## References

[1] Gosmanov AR Umpierrez GE. Management of hyperglycemia during enteral and parenteral nutrition therapy. Curr Diab Rep. (2013) 13:155–62. 10.1007/s11892-012-0335-y 23065369 PMC3746491

[2] Umpierrez GE Isaacs SD Bazargan N You X Thaler LM Kitabchi AE. Hyperglycemia: an independent marker of in-hospital mortality in patients with undiagnosed diabetes. J Clin Endocrinol Metab. (2002) 87:978–82. 10.1210/jcem.87.3.8341 11889147

[3] Cook CB Kongable GL Potter DJ Abad VJ Leija DE Anderson M. Inpatient glucose control: a glycemic survey of 126 US hospitals. J Hosp Med. (2009) 4:E7–14. 10.1002/jhm.533 20013863

[4] Cook A Burkitt D Mcdonald L Sublett L. Evaluation of glycemic control using NPH insulin sliding scale versus insulin aspart sliding scale in continuously tube-fed patients. Nutr Clin Pract. (2009) 24:718–22. 10.1177/0884533609351531 19955549

[5] Pleva M Mirtallo JM Steinberg SM. Hyperglycemic events in non–intensive care unit patients receiving parenteral nutrition. Nutr Clin Pract. (2009) 24:626–34. 10.1177/0884533609339069 19564627

[6] Furnary AP Gao G Grunkemeier GL Wu Y Zerr KJ Bookin SO. Continuous insulin infusion reduces mortality in patients with diabetes undergoing coronary artery bypass grafting. J Thorac Cardiovasc Surg. (2003) 125:1007–21. 10.1067/mtc.2003.181 12771873

[7] Jenkins DJ Wolever T Taylor RH Barker H Fielden H Baldwin JM. Glycemic index of foods: a physiological basis for carbohydrate exchange. Am J Clin Nutr. (1981) 34:362–6. 10.1093/ajcn/34.3.362 6259925

[8] Schwingshackl L Chaimani A Hoffmann G Schwedhelm C Boeing H. Impact of different dietary approaches on glycemic control and cardiovascular risk factors in patients with type 2 diabetes: a protocol for a systematic review and network meta-analysis. Syst Rev. (2017) 6:1–7. 10.1186/s13643-017-0455-1 28320464 PMC5360012

[9] Sheard NF Clark NG Brand-Miller JC Franz MJ Pi-Sunyer FX Mayer-Davis E. Dietary carbohydrate (amount and type) in the prevention and management of diabetes: a statement by the American Diabetes Association. Diabetes Care. (2004) 27:2266–71. 10.2337/diacare.27.9.2266 15333500

[10] Collier G O'dea K. The effect of coingestion of fat on the glucose, insulin, and gastric inhibitory polypeptide responses to carbohydrate and protein. Am J Clin Nutr. (1983) 37:941–44. 10.1093/ajcn/37.6.941 6342357

[11] Collier G Mclean A O'dea K. Effect of co-ingestion of fat on the metabolic responses to slowly and rapidly absorbed carbohydrates. Diabetologia. (1984) 26:50–4. 10.1007/BF00252263 6368300

[12] Nuttall FQ Gannon MC. Plasma glucose and insulin response to macronutrients in nondiabetic and NIDDM subjects. Diabetes Care. (1991) 14:824–38. 10.2337/diacare.14.9.824 1959475

[13] Floyd JC Fajans SS Pek S Thiffault CA Knopf RF Conn JW. Synergistic effect of essential amino acids and glucose upon insulin secretion in man. Diabetes. (1970) 19:109–15. 10.2337/diab.19.2.109 4905636

[14] Nuttall FQ Mooradian AD Gannon MC Billington C Krezowski P. Effect of protein ingestion on the glucose and insulin response to a standardized oral glucose load. Diabetes Care. (1984) 7:465–70. 10.2337/diacare.7.5.465 6389060

[15] Van Loon LJ Saris WH Verhagen H Wagenmakers AJ. Plasma insulin responses after ingestion of different amino acid or protein mixtures with carbohydrate. Am J Clin Nutr. (2000) 72:96–105. 10.1093/ajcn/72.1.96 10871567

[16] Doaei S Kalantari N Izadi P Salonurmi T Mosavi Jarrahi A Rafieifar S. Changes in FTO and IRX3 gene expression in obese and overweight male adolescents undergoing an intensive lifestyle intervention and the role of FTO genotype in this interaction. J Transl Med. (2019) 17:176. 10.1186/s12967-019-1921-4 31126299 PMC6534854

[17] Hong X Xu F Wang Z Liang Y Li J. Dietary patterns and the incidence of hyperglyacemia in China. Public Health Nutr. (2016) 19:131–41. 10.1017/S1368980015000774 25880390 PMC10270941

[18] Ashkar F Rezaei S Salahshoornezhad S Vahid F Gholamalizadeh M Dahka SM. The Role of medicinal herbs in treatment of insulin resistance in patients with polycystic ovary syndrome: a literature review. Biomol Concepts. (2020) 11:57–75. 10.1515/bmc-2020-0005 32229652

[19] Vahid F Hekmatdoost A Mirmajidi S Doaei S Rahmani D Faghfoori Z. Association between index of nutritional quality and nonalcoholic fatty liver disease: the role of vitamin D and B group. Am J Med Sci. (2019) 358:212–8. 10.1016/j.amjms.2019.06.008 31326093

[20] Feskens EJ Virtanen SM Räsänen L Tuomilehto J Stengård J Pekkanen J. Dietary factors determining diabetes and impaired glucose tolerance: a 20-year follow-up of the Finnish and Dutch cohorts of the seven countries study. Diabetes Care. (1995) 18:1104–12. 10.2337/diacare.18.8.1104 7587845

[21] Narasimhan S Nagarajan L Vaidya R Gunasekaran G Rajagopal G Parthasarathy V. Dietary fat intake and its association with risk of selected components of the metabolic syndrome among rural South Indians. Indian J Endocrinol Metab. (2016) 20:47. 10.4103/2230-8210.172248 26904468 PMC4743383

[22] Hu FB Van Dam R Liu S. Diet and risk of type II diabetes: the role of types of fat and carbohydrate. Diabetologia. (2001) 44:805–17. 10.1007/s001250100547 11508264

[23] Meyer KA Kushi LH Jacobs DR Folsom AR. Dietary fat and incidence of type 2 diabetes in older Iowa women. Diabetes Care. (2001) 24:1528–35. 10.2337/diacare.24.9.1528 11522694

[24] Salmeron J Hu FB Manson JE Stampfer MJ Colditz GA Rimm EB. Dietary fat intake and risk of type 2 diabetes in women. Am J Clin Nutr. (2001) 73:1019–26. 10.1093/ajcn/73.6.1019 11382654

[25] Guasch-Ferré M Becerra-Tomas N Ruiz-Canela M Corella D Schröder H Estruch R. Total and subtypes of dietary fat intake and risk of type 2 diabetes mellitus in the Prevención con Dieta Mediterránea (PREDIMED) study. Am J Clin Nutr. (2017) 105, 723–735. 10.3945/ajcn.116.142034 28202478

[26] Behrad Nasab M Afsharfar M Ahmadzadeh M Vahid F Gholamalizadeh M Abbastorki S. Comparison of the index of nutritional quality in breast cancer patients with healthy women. Front Nutr. (2022) 9:811827. 10.3389/fnut.2022.811827 35399658 PMC8989282

[27] Asghari G Mirmiran P Rashidkhani B Asghari-Jafarabadi M Mehran M Azizi F. The association between diet quality indices and obesity: tehran lipid and glucose study. Arch Iran Med. (2012) 15:599–605. 23020534

[28] Asghari G Mirmiran P Hosseni-Esfahani F Nazeri P Mehran M Azizi F. Dietary quality among Tehranian adults in relation to lipid profile: findings from the Tehran lipid and glucose study. J Health Popul Nutr. (2013) 31:37–48. 10.3329/jhpn.v31i1.14747 23617203 PMC3702357

[29] Daneshzad E Larijani B Azadbakht L. Diet quality indices and cardiovascular diseases risk factors among diabetic women. J Sci Food Agric. (2019) 99:5926–33. 10.1002/jsfa.9867 31206677

[30] Lassale C Gunter MJ Romaguera D Peelen LM Van Der Schouw YT Beulens JW. Diet quality scores and prediction of all-cause, cardiovascular and cancer mortality in a pan-European cohort study. PLoS ONE. (2016) 11:e0159025. 10.1371/journal.pone.0159025 27409582 PMC4943719

[31] Gopinath B Rochtchina E Flood V Mitchell P. Diet quality is prospectively associated with incident impaired fasting glucose in older adults. Diabetic medicine. (2013) 30:557–62. 10.1111/dme.12109 23301551

[32] Vahid F Rahmani D Davoodi SH Hekmatdoost A. The association among maternal index of nutritional quality, dietary antioxidant index, and odds of miscarriage incidence: case-control study. J Am Coll Nutr. (2022) 41:310–7. 10.1080/07315724.2021.1880987 33783310

[33] Gholamalizadeh M Rastgoo S Doaei S Vahid F Malmir H Ashoori N. Index of nutritional quality (INQ) and the risk of obesity in male adolescents: a case-control study. Biol Trace Elem Res. (2021) 199:1701–6. 10.1007/s12011-020-02297-3 32895892

[34] Horváth M Babinszky L. Impact of selected antioxidant vitamins (Vitamin A, E and C) and micro minerals (Zn, Se) on the antioxidant status and performance under high environmental temperature in poultry: A review. Acta Agriculturae Scandinavica. (2018) 68:152–60. 10.1080/09064702.2019.1611913

[35] Meerza D Iqbal S Zaheer S Naseem I. Retinoids have therapeutic action in type 2 diabetes. Nutrition. (2016) 32:898–903. 10.1016/j.nut.2016.02.003 27134203

[36] Iqbal S Naseem I. Role of vitamin A in type 2 diabetes mellitus biology: effects of intervention therapy in a deficient state. Nutrition. (2015) 31:901–7. 10.1016/j.nut.2014.12.014 26001806

[37] Shidfar F Aghasi M Vafa M Heydari I Hosseini S Shidfar S. Effects of combination of zinc and vitamin A supplementation on serum fasting blood sugar, insulin, apoprotein B and apoprotein AI in patients with type I diabetes. Int J Food Sci Nutr. (2010) 61:182–91. 10.3109/09637480903334171 20151940

[38] Jafarirad S Siassi F Harirchian M-H Amani R Bitarafan S Saboor-Yaraghi A. The effect of vitamin a supplementation on biochemical parameters in multiple sclerosis patients. Iran Red Crescent Med J. (2013) 15:194. 10.5812/ircmj.3480 23983997 PMC3745746

[39] De Lourdes Lima M Cruz T Pousada JC Rodrigues LE Barbosa K Canguçu V. The effect of magnesium supplementation in increasing doses on the control of type 2 diabetes. Diabetes Care. (1998) 21:682–6. 10.2337/diacare.21.5.682 9589224

[40] De Valk H. Verkaaik R. Van Rijn H. Geerdink R. Struyvenberg A. (1998). Oral magnesium supplementation in insulin-requiring Type 2 diabetic patients. Diabetic Med. 15, 503–7. 10.1002/(SICI)1096-9136(199806)15:6<503::AID-DIA596>3.0.CO;2-M 9632126

[41] Rodríguez-Morán M Guerrero-Romero F. Oral magnesium supplementation improves insulin sensitivity and metabolic control in type 2 diabetic subjects: a randomized double-blind controlled trial. Diabetes Care. (2003) 26:1147–52. 10.2337/diacare.26.4.1147 12663588

[42] Solati M Ouspid E Hosseini S Soltani N Keshavarz M Dehghani M. Oral magnesium supplementation in type II diabetic patients. Med J Islam Repub Iran. (2014) 28:67. 25405132 PMC4219896

[43] Paolisso G Barbagallo M. Hypertension, diabetes mellitus, and insulin resistance: the role of intracellular magnesium. Am J Hypertens. (1997) 10:346–55. 10.1016/S0895-7061(96)00342-1 9056694

[44] Takaya J Higashino H Kobayashi Y. Intracellular magnesium and insulin resistance. Magnesium Res. (2004) 17:126–36. 15319146

[45] Kolterman O Gray R Griffin J Burstein P Insel J Scarlett J. Receptor and postreceptor defects contribute to the insulin resistance in noninsulin-dependent diabetes mellitus. J Clin Invest. (1981) 68:957–69. 10.1172/JCI110350 7287908 PMC370882

[46] Fang L Li X. Level of serum phosphorus and adult type 2 diabetes mellitus. J Cent South Univ Med Sci. (2016) 41:502–6. 10.11817/j.issn.1672-7347.2016.05.009 27269925

[47] Duan S Sun L Zhu H Nie G Zhang C Huang Z. Association of urinary calcium and phosphorus excretion with renal disease progression in type 2 diabetes. Diabetes Res Clin Pract. (2021) 178:108981. 10.1016/j.diabres.2021.108981 34311020

[48] Vorum H Ditzel J. Disturbance of inorganic phosphate metabolism in diabetes mellitus: its relevance to the pathogenesis of diabetic retinopathy. J Ophthalmol. (2014) 2014:135287. 10.1155/2014/135287 24782919 PMC3980928

[49] Babu PS Srinivasan K. Influence of dietary capsaicin and onion on the metabolic abnormalities associated with streptozotocin induced diabetes mellitus. Mol Cell Biochem. (1997) 175:49–57. 10.1023/A:1006881027166 9350033

[50] Ditzel J Lervang H-H. Disturbance of inorganic phosphate metabolism in diabetes mellitus: its impact on the development of diabetic late complications. Curr Diabetes Rev. (2010) 6:323–33. 10.2174/157339910793360833 20701584

[51] Farooq DM Alamri AF Alwhahabi BK Metwally AM Kareem KA. The status of zinc in type 2 diabetic patients and its association with glycemic control. J Family Community Med. (2020) 27:29. 10.29309/TPMJ/2020.27.10.4048 32030076 PMC6984028

[52] Chausmer AB. Zinc, insulin and diabetes. J Am Coll Nutr. (1998) 17:109–15. 10.1080/07315724.1998.10718735 9550453

[53] Saharia GK Goswami RK. Evaluation of serum zinc status and glycated hemoglobin of type 2 diabetes mellitus patients in a tertiary care hospital of assam. J Lab Physicians. (2013) 5:30–3. 10.4103/0974-2727.115923 24014965 PMC3758701

[54] Masood N Baloch GH Ghori RA Memon IA Memon MA Memon MS. Serum zinc and magnesium in type-2 diabetic patients. J Coll Physicians Surg Pak. (2009) 19:483–6. 19651009

[55] Salgueiro MJ Krebs N Zubillaga MB Weill R Postaire E Lysionek AE. Zinc and diabetes mellitus. Biol Trace Elem Res. (2001) 81:215–28. 10.1385/BTER:81:3:215 11575679

[56] Wijesekara N Chimienti F Wheeler M. Zinc, a regulator of islet function and glucose homeostasis. Diabetes Obes Metab. (2009) 11:202–14. 10.1111/j.1463-1326.2009.01110.x 19817803

[57] Zwakenberg SR Remmelzwaal S Beulens JW Booth SL Burgess S Dashti HS. Circulating phylloquinone concentrations and risk of type 2 diabetes: a mendelian randomization study. Diabetes. (2019) 68:220–5. 10.2337/db18-0543 30352877 PMC6314462

[58] Beulens JW Grobbee DE Sluijs I Spijkerman AM Van Der Schouw YT. Dietary phylloquinone and menaquinones intakes and risk of type 2 diabetes. Diabetes Care. (2010) 33:1699–705. 10.2337/dc09-2302 20424220 PMC2909045

[59] Ibarrola-Jurado N Salas-Salvado J Martinez-Gonzalez MA Bullo M. Dietary phylloquinone intake and risk of type 2 diabetes in elderly subjects at high risk of cardiovascular disease. Am J Clin Nutr. (2012) 96:1113–8. 10.3945/ajcn.111.033498 23034962

[60] Comerford KB. Recent developments in multivitamin/mineral research. Adv Nutr. (2013) 4:644–56. 10.3945/an.113.004523 24228193 PMC3823510

[61] Reddi K Henderson B Meghji S Wilson M Poole S Hopper C. Interleukin 6 production by lipopolysaccharide-stimulated human fibroblasts is potently inhibited by naphthoquinone (vitamin K) compounds. Cytokine. (1995) 7:287–90. 10.1006/cyto.1995.0034 7640347

[62] Ohsaki Y Shirakawa H Hiwatashi K Furukawa Y Mizutani T Komai M. Vitamin K suppresses lipopolysaccharide-induced inflammation in the rat. Biosci Biotechnol Biochem. (2006) 70:926–32. 10.1271/bbb.70.926 16636460

[63] Shea MK Booth SL Massaro JM Jacques PF D'Agostino RB Sr Dawson-Hughes B. Vitamin K and vitamin D status: associations with inflammatory markers in the Framingham Offspring Study. Am J Epidemiol. (2008) 167:313–20. 10.1093/aje/kwm306 18006902 PMC3151653

[64] Sikkens EC Cahen DL Koch AD Braat H Poley J-W Kuipers EJ. The prevalence of fat-soluble vitamin deficiencies and a decreased bone mass in patients with chronic pancreatitis. Pancreatology. (2013) 13:238–42. 10.1016/j.pan.2013.02.008 23719594

[65] Tan M Sheng L Qian Y Ge Y Wang Y Zhang H. Changes of serum selenium in pregnant women with gestational diabetes mellitus. Biol Trace Elem Res. (2001) 83:231–7. 10.1385/BTER:83:3:231 11794515

[66] Al-Saleh E Nandakumaran M Al-Shammari M Al-Harouny A. Maternal–fetal status of copper, iron, molybdenum, selenium and zinc in patients with gestational diabetes. J Matern Fetal Neonatal Med. (2004) 16:15–21. 10.1080/14767050412331283139 15370077

[67] Bo S Lezo A Menato G Gallo M-L Bardelli C Signorile A. Gestational hyperglycemia, zinc, selenium, and antioxidant vitamins. Nutrition. (2005) 21:186–91. 10.1016/j.nut.2004.05.022 15723747

[68] Burlet E Jain SK. Manganese supplementation reduces high glucose-induced monocyte adhesion to endothelial cells and endothelial dysfunction in Zucker diabetic fatty rats. J Biol Chem. (2013) 288:6414–09. 10.1074/jbc.M112.447805 23329836 PMC3585075

[69] Juttukonda LJ Berends ETM Zackular JP Moore JL Stier MT Zhang Y. Dietary manganese promotes staphylococcal infection of the heart. Cell Host Microbe. (2017) 22: 531–42.e8. 10.1016/j.chom.2017.08.009 28943329 PMC5638708

[70] Gong JH Lo K Liu Q Li J Lai S Shadyab AH. Dietary manganese, plasma markers of inflammation, and the development of type 2 diabetes in postmenopausal women: findings from the women's health initiative. Diabetes Care. (2020) 43:1344–51. 10.2337/dc20-0243 32295807 PMC7245351

[71] Shan Z Chen S Sun T Luo C Guo Y Yu X. U-shaped association between plasma manganese levels and type 2 diabetes. Environ Health Perspect. (2016) 124:1876–81. 10.1289/EHP176 27258818 PMC5132633

[72] Aschner JL Aschner M. Nutritional aspects of manganese homeostasis. Mol Aspects Med. (2005) 26:353–62. 10.1016/j.mam.2005.07.003 16099026 PMC6309959

[73] Li L Yang X. The essential element manganese, oxidative stress, and metabolic diseases: links and interactions. Oxid Med Cell Longev. (2018) 2018:7580707. 10.1155/2018/7580707 29849912 PMC5907490

